# Assessing spatial and temporal biases and gaps in the publicly available distributional information of Iberian mosses

**DOI:** 10.3897/BDJ.8.e53474

**Published:** 2020-09-15

**Authors:** Cristina Ronquillo, Fernanda Alves-Martins, Vicente Mazimpaka, Thadeu Sobral-Souza, Bruno Vilela-Silva, Nagore G. Medina, Joaquín Hortal

**Affiliations:** 1 Dept. Biogeography & Global Change, Museo Nacional de Ciencias Naturales (MNCN-CSIC), Madrid, Spain Dept. Biogeography & Global Change, Museo Nacional de Ciencias Naturales (MNCN-CSIC) Madrid Spain; 2 Dept. Biología (Botánica), Facultad de Ciencias, Universidad Autónoma de Madrid, Madrid, Spain Dept. Biología (Botánica), Facultad de Ciencias, Universidad Autónoma de Madrid Madrid Spain; 3 Dept. Botanica e Ecologia, Universidade Federal de Mato Grosso (UFMT), Cuiaba, Brazil Dept. Botanica e Ecologia, Universidade Federal de Mato Grosso (UFMT) Cuiaba Brazil; 4 Instituto de Biologia, Universidade Federal da Bahia. 1154, R. Barão de Jeremoabo, 668 - Ondina, Salvador, Brazil Instituto de Biologia, Universidade Federal da Bahia. 1154, R. Barão de Jeremoabo, 668 - Ondina Salvador Brazil; 5 Universidade Federal de Goiás, Goiânia, Brazil Universidade Federal de Goiás Goiânia Brazil; 6 Faculdade de Ciências da Universidade de Lisboa, Lisboa, Portugal Faculdade de Ciências da Universidade de Lisboa Lisboa Portugal

**Keywords:** Biodiversity data, Bryophyta, Global Biodiversity Information Facility, IberBryo, Iberian Peninsula, Inventory completeness, Wallacean Shortfall

## Abstract

One of the most valuable initiatives on massive availability of biodiversity data is the Global Biodiversity Information Facility, which is creating new opportunities to develop and test macroecological knowledge. However, the potential uses of these data are limited by the gaps and biases associated to large-scale distributional databases (the so-called Wallacean shortfall). Describing and quantifying these limitations are essential to improve knowledge on biodiversity, especially in poorly-studied groups, such as mosses. Here we assess the coverage of the publicly-available distributional information on Iberian mosses, defining its eventual biases and gaps. For this purpose, we compiled IberBryo v1.0, a database that comprises 82,582 records after processing and checking the geospatial and taxonomical information. Our results show the limitations of data and metadata of the publicly-available information. Particularly, ca. 42% of the records lacked collecting date information, which limits data usefulness for time coverage analyses and enlarges the existing knowledge gaps. Then we evaluated the overall coverage of several aspects of the spatial, temporal and environmental variability of the Iberian Peninsula. Through this assessment, we demonstrate that the publicly-available information on Iberian mosses presents significant biases. Inventory completeness is strongly conditioned by the recorders' survey bias, particularly in northern Portugal and eastern Spain and the spatial pattern of surveys is also biased towards mountains. Besides, the temporal pattern of survey effort intensifies from 1970 onwards, encompassing a progressive increase in the geographic coverage of the Iberian Peninsula. Although we just found 5% of well-surveyed cells of 30’ of resolution over the 1970-2018 period, they cover about a fifth of the main climatic gradients of the Iberian Peninsula, which provides a fair – though limited – coverage. Yet, the well-surveyed cells are biased towards anthropised areas and some of them are located in areas under intense land-use changes, mainly due to the wood-fires of the last decade. Despite the overall increase, we found a noticeable gap of information in the south-west of Iberia, the Ebro river basin and the inner plateaus. All these gaps and biases call for a careful use of the available distributional data of Iberian mosses for biogeographical and ecological modelling analysis. Further, our results highlight the necessity of incorporating several good practices to increase the coverage of high-quality information. These good practices include digitalisation of specimens and metadata information, improvement on the protocols to get accurate data and metadata or revisions of the vouchers and recorders' field notebooks. These procedures are essential to improve the quality and coverage of the data. Finally, we also encourage Iberian bryologists to establish a series of re-surveys of classical localities that would allow updating the information on the group, as well as to design their future surveys considering the most important information gaps on IberBryo.

## Introduction

The current massive availability of biodiversity data is creating new opportunities to develop and test macroecological knowledge (Hampton et al. 2013, Morueta-Holme and Svenning 2018). Advances in the management (i.e. acquisition, cleaning and integration) and analysis of ‘biodiversity big data’ are crucial (Gandomi and Haider 2015, Devictor and Bensaude-Vincent 2016), thus promoting the emergence of new fields such as eco-informatics and biodiversity informatics (Bisby 2000, Soberón and Peterson 2004). One of the most valuable initiatives on this matter is the Global Biodiversity Information Facility (GBIF, http://www.gbif.org/), a distributed network of databases that seeks to provide open access to all biodiversity data through the internet (Saarenmaa and Nielsen 2002). The GBIF platform offers a vast amount of primary distributional information that allows outlining large-scale questions from a data-driven approach (García-Roselló et al. 2015, Franklin et al. 2017).

Advances in big biodiversity data tools and computational power are continually increasing the potential offered by this information (Bisby 2000, Maldonado et al. 2015, Devictor and Bensaude-Vincent 2016, Wüest et al. 2019). However, managing the vast amount of data is challenging due to its large volume and the high variability, velocity and variety in the creation, veracity and value of data (Gandomi and Haider 2015, Devictor and Bensaude-Vincent 2016, Wüest et al. 2019). Data pre-processing is key to reach adequate levels of quality and reliability of the records that are finally analysed (Calabrese 2019). The more common limitations of biodiversity data are related to georeferencing and taxonomy (Soberón and Peterson 2004, Wieczorek et al. 2004, Yesson et al. 2007, Sousa-Baena et al. 2014, Isaac and Pocock 2015) and data cleaning processes have an important role in their solution (Chapman 2005, Gandomi and Haider 2015, Maldonado et al. 2015, Gueta and Carmel 2016, Calabrese 2019).

Once these issues are handled, the subsequent task would be to assess the quality of data as a whole. In the particular case of macroecology and biogeography, this means addressing the gaps and biases associated to large-scale databases (Hortal et al. 2007, Beck et al. 2013, Engemann et al. 2015, Amano et al. 2016, Meyer et al. 2016), which compromise the description of biodiversity patterns (Hortal et al. 2008, Boakes et al. 2010, Yang et al. 2013, Beck et al. 2014, Hortal et al. 2015, Morueta-Holme and Svenning 2018). By evaluating and describing how these limitations affect the geographic distribution of species – the so-called Wallacean shortfall (Lomolino 2004) – it is possible to enhance the insights obtained with these data and also design research seeking to fill in the gaps in this knowledge (Rocchini et al. 2011, Hortal et al. 2015, Morueta-Holme and Svenning 2018, Wetzel et al. 2018). Essentially, the Wallacean shortfall is due to uneven sampling effort through space and time, typically caused by the historical patterns of collecting and analysing data (Hortal et al. 2007, Hortal et al. 2008, Sastre and Lobo 2009, Hortal et al. 2015, Isaac and Pocock 2015, Maldonado et al. 2015, Amano et al. 2016). To overcome this shortfall, we need to evaluate and quantify the survey coverage of biodiversity data along space, time, environment and taxonomy (Hortal et al. 2008, Boakes et al. 2010, Meyer et al. 2015, Meyer et al. 2016, Troia and McManamay 2016).

The extent of the Wallacean shortfall varies considerably amongst taxonomic groups (Amano et al. 2016, Troia and McManamay 2016), depending on the historical interest on the survey or study of each one of them. While the study of diversity patterns at large scales has been mainly focused on vascular plants and vertebrates (Mutke and Barthlott 2005, Aranda et al. 2015), bryophytes have been considered just on a few occasions (Mutke and Geffert 2010, Geffert et al. 2013, Hespanhol et al. 2015, Mateo et al. 2016, Berdugo et al. 2018). Therefore, although the knowledge on this highly-diverse group of organisms has been developed over a long historical period (Magill 2010), especially in Europe (Mutke and Geffert 2010), the quality of moss distributional data has been scarcely assessed (Callaghan and Ashton 2008, Mutke and Geffert 2010, Aranda et al. 2011, Meyer et al. 2016). As a result, the coverage of its spatial and temporal distributional information is poorly-known and may, indeed, reflect the historical pattern of surveys, rather than the actual diversity of this group (Mutke and Barthlott 2005).

Here we aim to assess and quantify the knowledge on the publicly-available distributional information on Iberian mosses, defining its eventual biases and gaps. To do this, we compile an extensive Iberian moss database, process its records to filter those with adequate quality and then analyse their coverage. Specifically we aim to: (i) assess the overall quality of moss records in the Iberian Peninsula; (ii) evaluate their substrate, altitudinal, temporal and spatial coverage; (iii) analyse their inventory completeness; and (iv) assess the adequacy of well-surveyed areas to recover the responses of biodiversity to climatic and land-use changes.

## Materials and Methods

### Pre-processing of occurrence data

**Figure 1.**
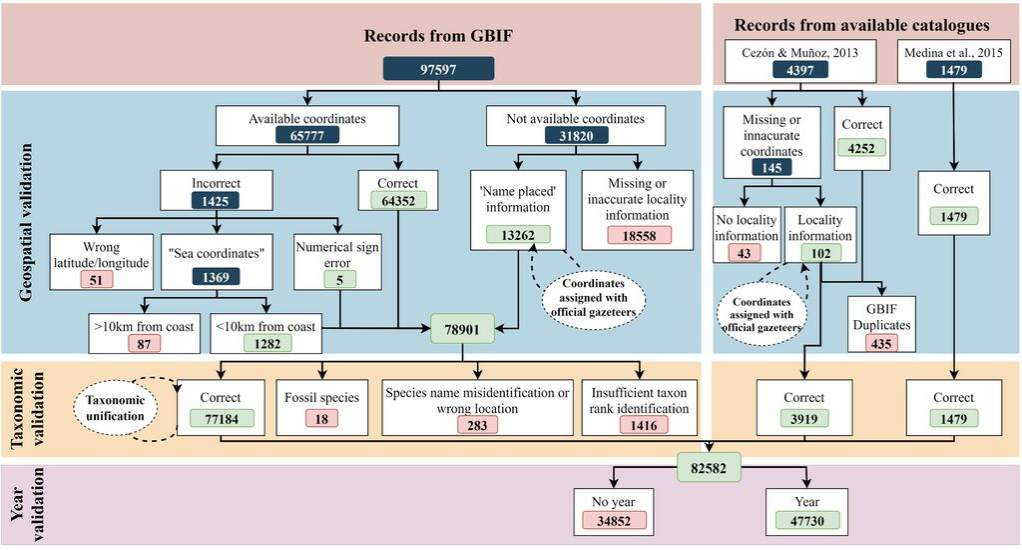
Pre-processing steps in the generation of IberBryo database and numbers of records managed in each one. Green numbers correspond to validated records and red numbers to deleted ones.

We downloaded 97,597 records of mosses (keyword Phylum: Bryophyta) for the Iberian Peninsula – defined as mainland Portugal and Spain, plus the Balearic Islands, Andorra and Gibraltar – from GBIF (GBIF 2018a, accessed 8 August 2018 for Spain and Portugal and GBIF 2018b, accessed 9 October 2018 for Andorra and Gibraltar). We also retrieved 5,876 occurrences from two PhD dissertations that comprised geographically-extensive surveys, encompassing several Spanish provinces and climatic zones (Cezón and Muñoz 2013, Medina et al. 2015). Records from Medina et al. (2015) – that include previously-surveyed areas in Galicia and Asturias from Albertos (2001) – were published in GBIF afterwards and they are now available in Medina and Ronquillo (2020). In total, the version 0.1 of our database (hereafter called IberBryo) held 103,473 unprocessed raw records. We will consider only good-quality occurrences for our analysis, i.e. those that represent an individual organism collected from certain location (i.e. latitude and longitude) and at a given time, such as, at least, calendar year (Troia and McManamay 2016; see also Hortal and Lobo 2005). In order to check and improve the quality of IberBryo records, we performed a data cleaning protocol (Fig. 1, Suppl. material 12) addressing the three main issues that may affect the quality of biological records: geospatial location, taxonomical identification and temporal allocation.

*Geospatial validation*. We checked the coordinates of all records following their available geographic location through ‘point-in-polygon’ test at province/district level with QGIS Development Team (2019) software and Global Administrative Areas (2018) country layers. Records that presented numerical sign errors were manually corrected, based on their locality description. Those placed on the sea, less than 10 km from the coast, were relocated at the nearest coastal place. Then, we georeferenced records without coordinates that presented a specific ‘named place’ (Wieczorek et al. 2004) in the locality description through geocoding using the corresponding official national gazetteers (as the geographic centre or locality centroids): “*Nomenclátor de Municipios y Entidades de población*” and “*Nomenclátor Básico*” of Instituto Geográfico Nacional (IGN) for Spanish records; “*Servicio de Localização Toponímica del Grupo Crise Rede de Informaçao de Situações de Emergencia*” for Portuguese records and “*Nomenclàtor Oficial del Govern d’Andorra*” for Andorran records. Finally, we discarded records lacking coordinates and outliers whose locality description was missing or inaccurate and those located on the sea more than 10 km from the coast.

*Taxonomic validation and standardisation.* We checked all species names (extracted from GBIF fields “scientific_name” and “genus” + “species”) to remove fossil specimens, misidentifications, wrong country locations or insufficient taxon rank identification. Records were reviewed following the checklists in Casas et al. (2006), Hill et al. (2006), Ros et al. (2013), Hodgetts (2015), Sotiaux and Vanderpoorten (2017) and *Flora Briofítica Ibérica* (Guerra et al. 2006, Brugués et al. 2007, Guerra et al. 2010, Guerra et al. 2014, Brugués and Guerra 2015, Guerra et al. 2018) under the expert supervision of one of us (VM). Subsequently, we unified the list of species names (correction of spelling, synonyms and authority standardisation) according to Hill et al. (2006) and Ros et al. (2013). For the assignation of the species name, we gave priority to the most recent checklist, except for taxa that have further experienced taxonomic or nomenclatural changes: for example, *Bartramia stricta* (Müller 2014), *Orthotrichum* (Plášek et al. 2015, Lara et al. 2016), *Codonoblepharon forsteri* (Goffinet et al. 2004, Mazimpaka and Lara 2014) and *Oxystegus tenuirostris* (Alonso et al. 2016, Alonso et al. 2018).

*Year validation*. We excluded all the occurrences without collecting date information at year level in the IberBryo v1.1 database to perform the climatic and land-use coverage analyses (see below), although we kept them in the IberBryo v1.0.

### Assessing survey coverage

Once all records had been pre-processed, we assessed the overall coverage of the spatial, temporal and environmental variability of the Iberian Peninsula provided by the inventories contained in IberBryo. All analyses were performed in R (R Development Core Team 2019 v 3.6) and RStudio (RStudio Team 2019 v 1.2) environment and coverage maps were customised in RWizard version 4.3 (Guisande et al. 2014). See the relation of scripts used in Suppl. material 13.

*Substrate coverage*. Due to the absence of habitat-type information in most of the records, we were only able to assess the coverage of ecological substrates by checking in specialised references all the taxa that thrive in each type of substrate. First, we made a simplified reclassification based on BRYOATT (Hill 2007), assigning each species to the following five substrate classes: rock, epiphytic, soil, aquatic and decaying vegetation. This reclassification includes information of the frequency of use for each species as follows: [1] Rare substrate [2] Occasional substrate [3] Normal substrate. Then, for taxa not included in this guide, the information was extracted from Dierssen (2001), Casas et al. (2006), Garilleti and Albertos (2012) and *Flora Briofítica Ibérica* (Guerra et al. 2006, Brugués et al. 2007, Guerra et al. 2010, Guerra et al. 2014, Brugués and Guerra 2015, Guerra et al. 2018).

*Altitudinal coverage*. We applied a Kolmogorov-Smirnov test to assess whether the altitudinal range, covered by moss occurrences, represented the altitudinal patterns of the study area. We attributed altitudinal data to each occurrence using a digital elevation model (DEM) of the study area at a spatial resolution of 30 arc-seconds, extracted from GMTED2010 (U.S. Geological Survey 2010) and the Iberian altitudinal patterns were calculated for all DEM data.

*Temporal coverage*. We represented the historical accumulation of new species (excluding infraspecific taxa) recorded in IberBryo and the number of records gathered by calendar years. Then we evaluated the relationship between number of records and newly-observed species per year through Spearman correlations. We defined different periods of data collection for the following analyses, based on the information provided by the curve and the main historical periods happening in the Iberian countries.

*Spatial coverage and survey completeness.* We calculated basic metrics of spatial coverage (number of records, observed richness and completeness) for all Iberian grid cells at two different resolutions, 5’ (~65 km^2^) and 30’ (~2500 km^2^), using the R package ‘KnowBR’ v 2.0 (Lobo et al. 2018). Metrics were calculated for each of the periods of data collection previously identified, as well as for the whole time series and the complete IberBryo v1.0 database (including occurrences without collecting date). We quantified inventory completeness in grid cells of 30’ resolution as a metric of survey quality coverage. Completeness for each grid cell was calculated by adjusting the species accumulation curve (i.e. accumulated number of species by records) to the Michaelis-Menten equation (Clench 1979, Soberón and Llorente 1993) and calculating the percentage of the moss flora of each cell predicted by the curve represented in the inventories (see Lobo et al. 2018). Cells with percentages of completeness higher than 80% and ≥ 10 records were considered as well-surveyed, while those with 70-80% were considered moderately well-surveyed and those with less than 70% as poorly-surveyed cells. These thresholds are arbitrary, based on our general knowledge on the survey process and our experience on surveying Iberian mosses. Therefore, qualifying cells as well-surveyed does not mean that their inventory is complete (or nearly complete), but rather that the species missing from the inventory are locally rare and/or inconspicuous. Although these absences are part of the moss assemblage of the grid cell, we assume that their importance for the diversity of local moss communities during the historical period represented by the surveys has been minimal.

We also obtained the location of the main bryology centres of Spain and Portugal. This selection was based on the more frequent affiliation centres collected on SCOPUS publications with the keywords “Bryophyte”, “moss”, “musgo” or “briofito”. We also extracted the location of recently-published PhD theses on bryophytes from Hespanhol et al. (2015) and checked the presence of this information in IberBryo. This allowed us to discuss and compare whether the spatial coverage results were biased by the spatial location of bryological research sources.

*Climatic coverage*. We assessed the coverage of the climatic variability of the Iberian Peninsula provided by the set of well-surveyed grid cells. To do this, we characterised the climatic environmental space of the Iberian Peninsula, based on the 19 bioclimatic variables from WorldClim 2.0 (Fick and Hijmans 2017) at 10’ resolution, aggregating them into the 30’ resolution cells of our study area. We performed a PCA to reduce the dimensionality of these data, obtaining two significant PCA axes that represent the main climatic gradients within Iberia and calculated the frequency of climate conditions in the Iberian Peninsula, based on the scores. Then, we quantified the overlap between the climatic space covered by the well-sampled cells and the climatic environmental space of the whole study area through the Schoener’s D index (Schoener 1974, Broennimann et al. 2012). Briefly, this index provides a measure of the overlap of two environmental envelopes, from 0 to 1 (complete overlap); in this particular case, Schoener's D value provides a measure of the proportion of the Iberian climatic variability covered by the well-sampled cells, as measured by the climatic PCA axes. We applied a Kolmogorov-Smirnov test to verify whether the distribution of climates shows statistically significant differences between all grid cells and well-sampled cells. We also quantified the ‘rarity index’ of these Iberian climate types as a ‘Min-Max scalling’. Based on their relative frequency, values are scaled from 0 — very common climate types — to 1 — very ‘rare’ or climatically unique. We also applied a Kolmogorov-Smirnov test to verify whether the distribution of well-sampled cells is biased to a certain climate type.

*Land-use change coverage*. We assessed the adequacy of moss data for representing changes in moss assemblages driven by recent land-use modifications in the Iberian Peninsula, following the method used for climatic coverage. We characterised recent land-use variations using information from *Corine Land Cover Changes* (Corine Land Cover seamless vector database- CLC v. 20; European Environmental Agency 2018) in different periods (1990-2000, 2000-2006, 2006-2012 and 2012-2018), available for Spain and Portugal. We reclassified the original CLC classes into simplest categories, according to the importance of each land-use type for bryophyte natural history (Suppl. material 11, Reclassification 1). We quantified the number of land use changes and their occupied area using the previous climatic grid of 30’ resolution cells from 1990 to 2018. We also assessed the ‘anthropised change ratio’ of the cells, based on a reclassification into artificial surfaces (‘Anthropic’) and natural surfaces (Suppl. material 11, Reclassification 2), as follows: ‘Anthropised only’ (Natural to Artificial); ‘Mostly anthropised’ (Natural to Artificial > Artificial to Natural); ‘Equally changed’ (Natural to Artificial = Artificial to Natural) and ‘Naturalised’ (Natural to Artificial < Artificial to Natural).

## Results

### Overall assessment of ‘IberBryo’ database

Version 1.0 of IberBryo database (Suppl. material 1; Ronquillo and Hortal 2020) includes 82,582 records after pre-processing validations, out of the 103,473 occurrences initially retrieved (Fig. 1). Only 57.80% (47,730) of these processed records include year information from 1783 to 2018 and, therefore, they comprise the bulk of IberBryo v1.1. We could retrieve 14.91% (14,549) of GBIF records mainly through the assignment of coordinates according to the locality description, while we had to delete 19.15% (18,696) of them due to geospatial errors (Fig. 1, see also Suppl. material 2). By countries, Spain contributes with most occurrences with year information (84.83%), followed by Portugal (14.75%) and Andorra (0.41%). There is only one record attributed to Gibraltar.

**Figure 2.**
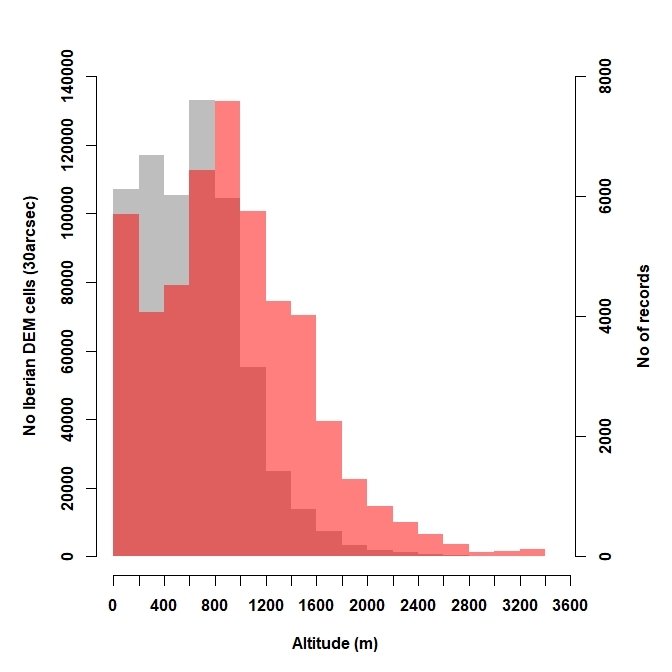
Altitudinal coverage of moss surveys, as the comparison between the altitudinal distributions of IberBryo v1.1 records (red bars) and the whole surface of the Iberian Peninsula (grey bars).

The taxonomic validation led to the deletion of 1,717 occurrences because of taxonomic issues (Fig. 1). Scientific names were unified in IberBryo v1.0 into 869 different species (including infraspecific taxa) from 57 families (857 out of 893 Spanish taxa, 369 out of 522 Portuguese taxa and 207 out of 274 Andorran taxa — totals extracted from Ros et al. 2013). Most of the species recorded in IberBryo are associated with rock and soil substrates (see Suppl. material 3). The altitudinal range covered by records of IberBryo v1.1 is biased towards high altitude places compared to the study area [Two-sample Kolmogorov-Smirnov test D = 0.272, p < 0.001] (Fig. 2).

**Figure 3.**
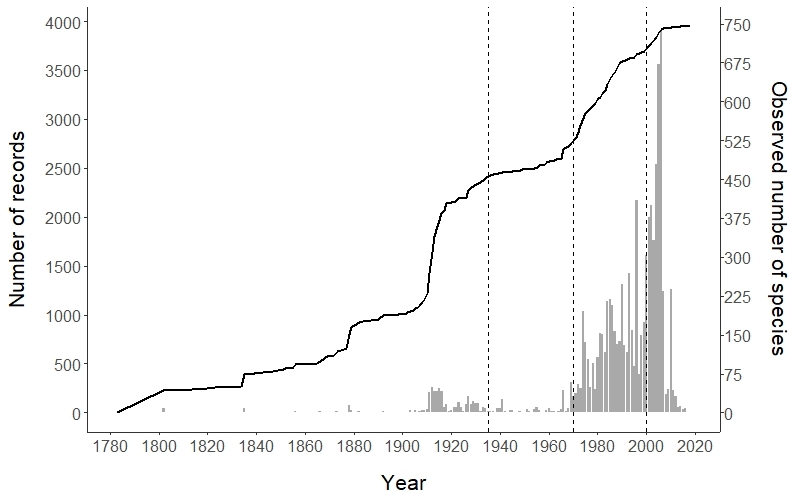
Historical progression of moss surveys in the Iberian Peninsula. Number of moss records gathered each year (grey bars) and accumulated number of species recorded in IberBryo (black line). Vertical dashed lines define different periods of historical data collection.

The historical pattern of moss surveys shows a steady increase in number of records and new species gathered through time. Due to the evaluation based on IberBryo v1.1 (only records with collecting date), the observed number of species (excluding infraspecific taxa) acummulated until 2018 was reduced to 745. The highest survey rates take place after 2000, and the accumulated number of observed species increased especially in the period 1970-1999 (Fig. 3). Records and number of species accumulated per year are strongly correlated through the whole time series (rho = 0.73, p < 0.001). Four distinct periods of collection — seemingly related to the political and overall academic situation of the Iberian countries — can be identified depending on changes in survey trends along the studied period: before 1935 (rho = 0.663, p < 0.001), 1936-1969 (rho = 0.256, p = 0.13), 1970-1999 (rho = 0.518, p = 0.003) and 2000-2018 (rho = -0.858, p < 0.001) (see Fig. 3).

### Spatial coverage and survey completeness

**Figure 4.**
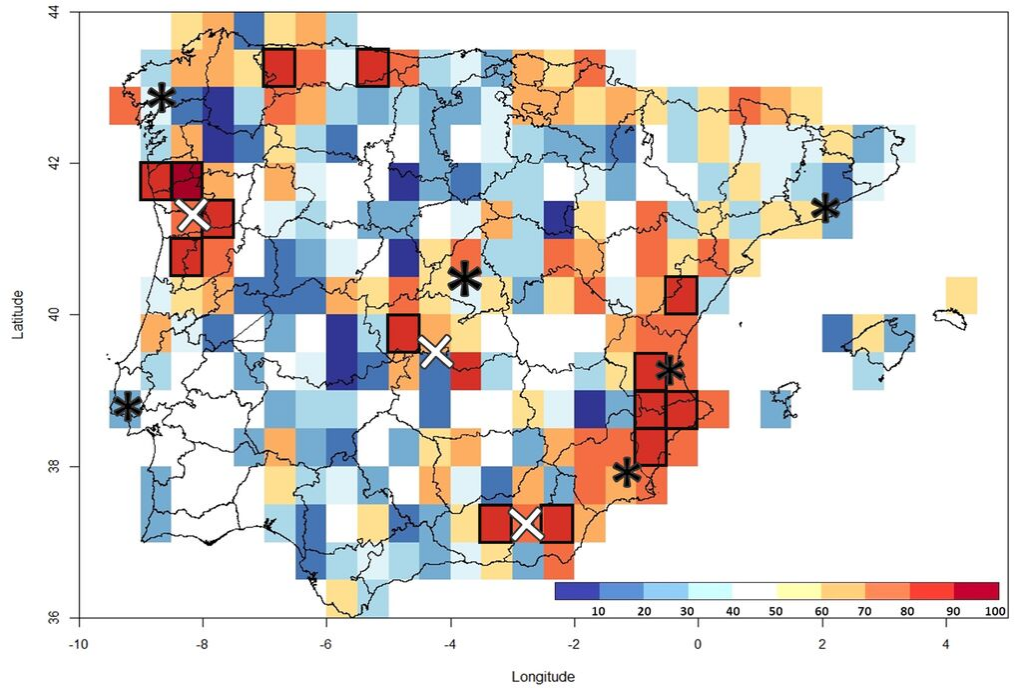
Geographic distribution of inventory completeness in the 1970-2018 period at 30’ resolution, according to the IberBryo v1.1 database. Values close to red represent higher percentages of completeness. Black squares correspond to well-surveyed cells (completeness ≥ 80% and number of records ≥ 10), white X-crosses to PhD theses – from left to right: Helena Hespanhol (NW Portugal), Katia Cezón (Castilla-La Mancha) and Susana Rams (Sierra Nevada) and black asterisks to major Iberian bryologist groups. These main research centres on bryophytes correspond to: Universidad Autónoma de Barcelona, Universidad Autónoma de Madrid, Universidad Complutense de Madrid, Universidade de Lisboa, Universidad de Murcia, Universidad Rey Juan Carlos, Universidade de Santiago de Compostela, Universitat de València, Museo Nacional de Ciencias Naturales (MNCN-CSIC) and Real Jardín Botánico (RJB-CSIC).

**Figure 5.**
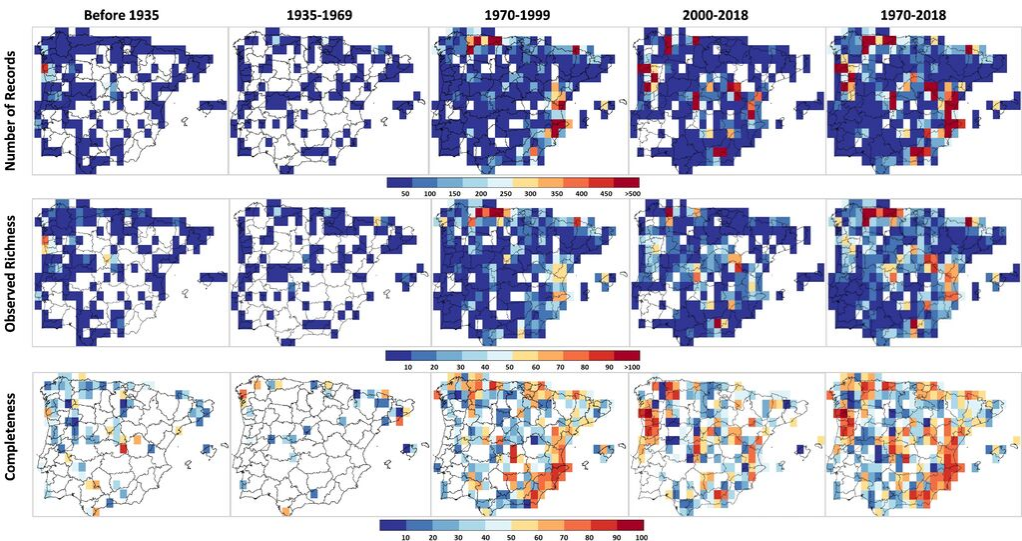
Geographical coverage of moss surveys along time in the Iberian Peninsula. Maps show the distribution of records numbers, observed richness and inventory completeness of Iberian mosses in each period at 30’ resolution in IberBryo v1.1.

**Figure 6.**
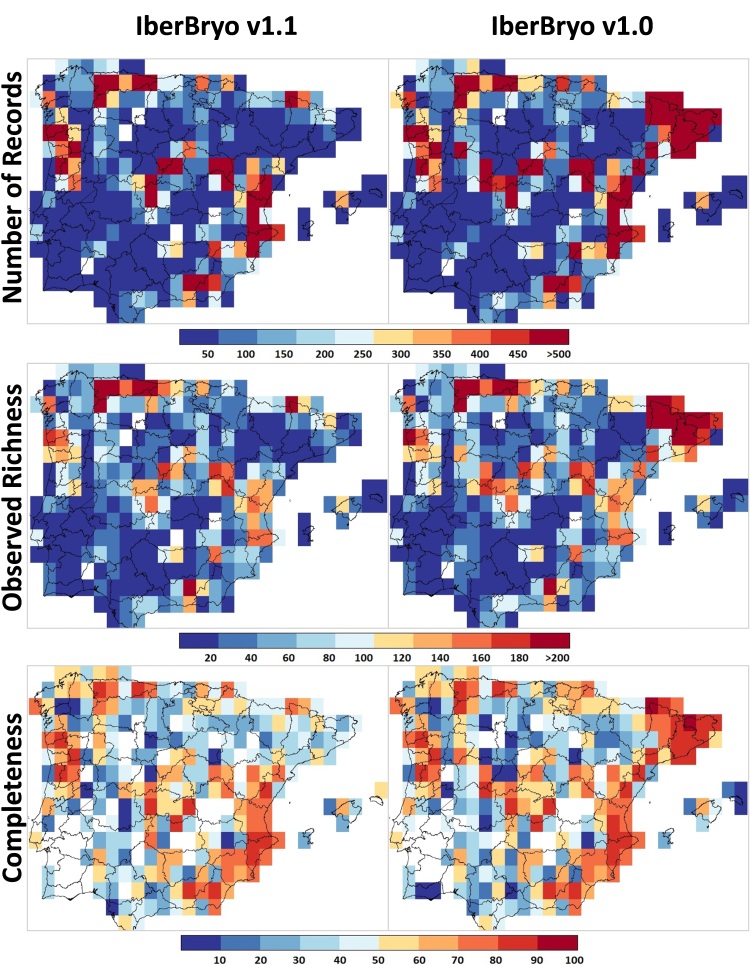
Geographical coverage of moss surveys as number of records, observed richness and inventory completeness included in IberBryo v1.1 database (with information on collecting date at year level; 1783-2018) and in IberBryo v1.0 database (including records without information on collecting date) at 30’ resolution.

The higher numbers of moss records, observed species richness and inventory completeness are mainly located in mountainous areas of the north and eastern Spanish coasts between 1970 and 1999 and in northern Portugal, central Spain and the mountainous area of Sierra Nevada between 2000 and 2018 (Fig. 5). Cells with very limited surveys or no information at all are located mainly in the inner plateaus and south-western Iberia, particularly after the year 2000 (Fig. 5). In the highly-surveyed period between 1970 and 2018, 4.98% of Iberian 30’ resolution cells (14 out of 281 cells) meet the criteria needed to be considered well-surveyed (Fig. 4), while only 0.36% (9 out of 2441 cells) do so at 5’ resolution (Suppl. material 6). An additional 8.9% of the 30' cells and 1.04% of the 5' cells were moderately-surveyed (25 and 26 cells, respectively). Considering the IberBryo v1.0 database, we find high levels in number of records, observed richness and completeness in north-eastern and north inner plateau of Spain with no information for the collecting date (Fig. 6). In addition, some of these cells present extremely high levels of survey completeness at 30’ resolution (Suppl. material 8), highlighting the potential value of these data if records’ information were complete.

### Environmental coverage

**Figure 7.**
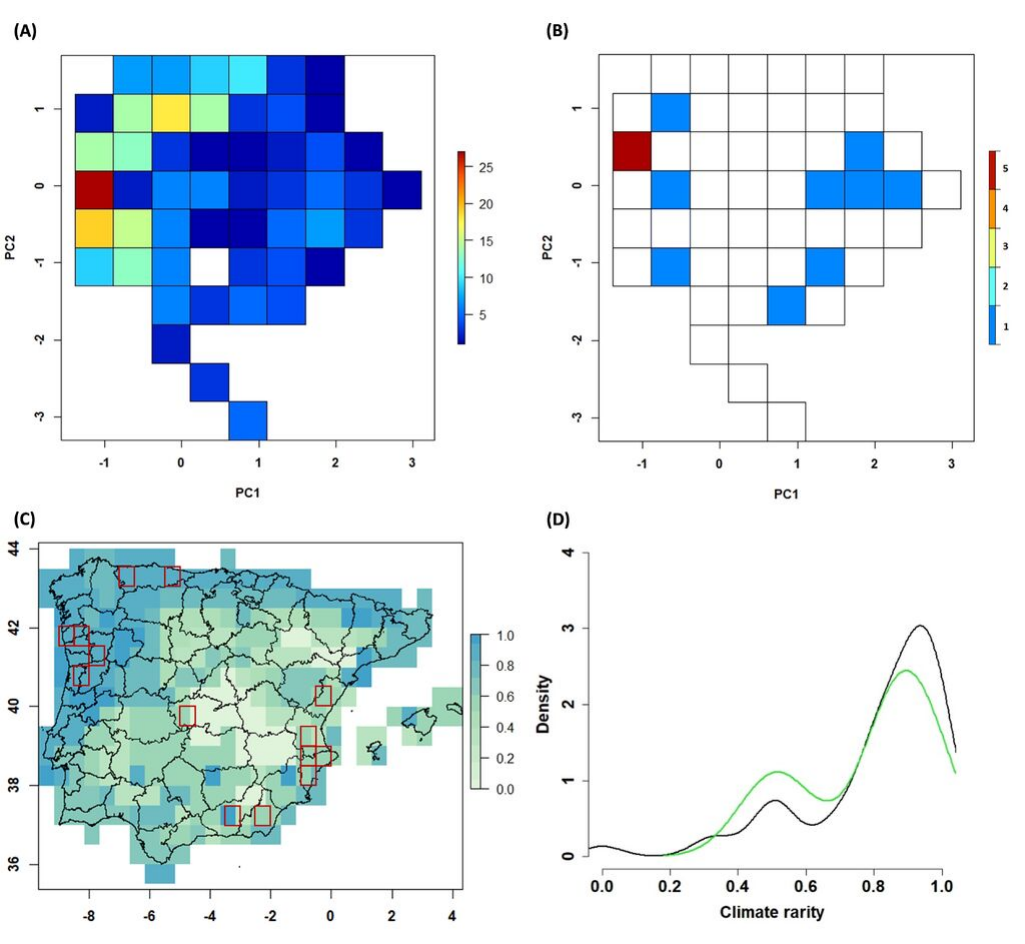
Climatic coverage of Iberian moss surveys. (A) Frequency of climate types in the Iberian environmental space (values indicate the number of 30’ cells of each climate type). (B) Frequency of climate types covered by well-surveyed cells (values indicate the number of 30’ cells of each climate type). (C) Geographic distribution of climatic rarity index in the study area (rarest climate types = 1), red squares indicate the location of well-surveyed moss cells. (D) Density comparison of the climatic rarity covered by Iberian cells (black line) and well-surveyed moss cells (green line).

**Figure 8.**
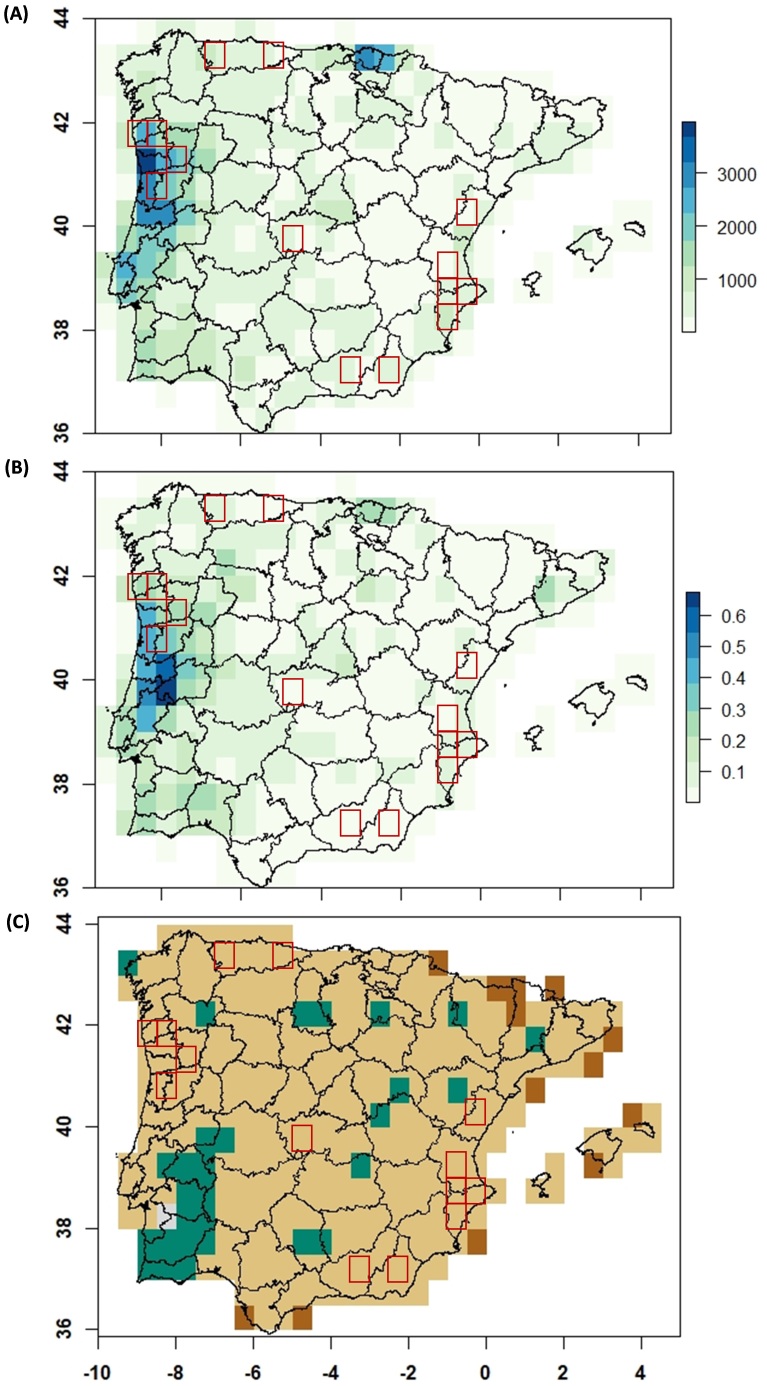
(A) Geographical distribution of frequency in land-use changes in 1990-2018 at 30’ resolution cells. (B) Proportion of land-use changed area in 1990-2018 at 30’ resolution cells. (C) Geographical distribution of ‘anthropised change ratio’ as artificial surfaces [A] or natural surfaces [N] changes. Dark brown cells ‘Anthropised only’ N to A; Light brown cells ‘Mostly anthropised’ N to A > A to N; Grey cells ‘Equally changed’ N to A = A to N; Green cells ‘Naturalised’ N to A < A to N. Red squares indicate the location of well-surveyed moss cells.

The PCA identified the two main gradients that characterise the climate of the Iberian Peninsula: one axis mainly related to seasonality — that separates the Mediterranean from Atlantic zones; and another axis related to temperature and (to a less extent) precipitation variations — that describes a gradient from cold (northern-mountainous) to warm-dry zones (central-south-eastern Iberia) (Suppl. material 9). We used these two axes to define an environmental space of 51 climate types at 30’ resolution (Fig. 7A), which captures 78% of the climate variability (Suppl. material 10). Well-surveyed cells cover 10 of these climate types (19.61%) (Fig. 7B), representing 21.75% of all climatic variability in the Iberian Peninsula (Schoener’s D = 0.218, p value = 0.002) (Suppl. material 9B). The coverage of the climatic variability occupied by well-surveyed moss cells is not biased in both axes when compared to the whole Iberian Peninsula: PC1 Two-sample Kolmogorov-Smirnov test D = 0.273, p = 0.272 and PC2 Two-sample Kolmogorov-Smirnov test D = 0.292, p = 0.203 (see also Suppl. material 9D). Well-surveyed cells also occupy more frequently ‘rare climatic conditions’ (Fig. 7C), but they show no differences compared to the distribution of climatic rarity in the Iberian Peninsula (Two-sample Kolmogorov-Smirnov test D = 0.176, p = 0.957) — which also present high levels of ‘rare climatic conditions’ (Fig. 7D). However, well-sampled areas provide a biased description of land-use changes across Iberia, as they are mostly placed in areas that have been changing towards higher proportions of artificial surfaces in the last decades, lacking data for cells that have followed naturalisation processes (Fig. 8C). Interestingly, the well-surveyed cells of northern Portugal are placed in the Iberian region with the highest rates of land use transformation (Fig. 8A).

## Discussion

Our analysis of the publicly-available data on Iberian mosses evidences the large extent of the shortfalls of the distributional information for this group. Besides, our study proves the crucial importance of data (and metadata) quality for evaluating the Wallacean shortfall for mosses, in the same way as has been established previously for other groups (Hortal et al. 2007, Yang et al. 2014, Meyer et al. 2016, Stropp et al. 2016). The diversity patterns of European mosses have been scarcely studied, at least when compared to flowering plants (Mutke and Barthlott 2005, Mutke and Geffert 2010, Geffert et al. 2013, Berdugo et al. 2018). The results above exposed evidence that the knowledge of such a common group with a long history of surveys in the Iberian Peninsula is, overall, insufficient. These surveys provide poor coverage of the distribution of moss diversity in this highly-heterogeneous region, which makes the assessment of its assemblage responses to climatic and land-use variations a challenging task. In fact, our results reveal that surveys are biased towards the location of the most important bryophyte researchers’ groups and mountainous areas. Issues on data quality, particularly the absence of information on collecting date, enlarge the existing biases even further. Despite these limitations, well-surveyed places are distributed throughout the whole study area. Indeed, they provide a fair (though limited) cover of about one fifth of the climate types of the Iberian Peninsula, which may allow using these data to model species and community responses to climate and assess the effects of climate change. Other aspects of global change are however less represented, because moss information is biased towards anthropised areas and some of the well-surveyed cells are located nearby an area that has suffered frequent land-use changes in the last decades.

The different biases, identified in moss biodiversity information, could compromise the reliability of eventual macroecological analysis carried out with the publicly-available data. Indeed, the main geographical pattern of observed species richness of Iberian mosses can be easily attributed to the recorders' home range (*sensu* Dennis and Thomas 2000; also known as taxonomist survey bias, Sastre and Lobo 2009). This is a common bias that has also been previously described for other groups (e.g. Lobo and Martin-Piera 2002, Oliveira et al. 2016, Girardello et al. 2019). In the case of Iberian mosses, well-surveyed areas and those with high density of records are placed near the bryologists’ homes and working places, especially in northern Portugal and eastern Spain, along with some exceptions determined by the particular location of PhD works. The spatial pattern of surveys also follows the relatively-common bias towards mountains, which results in a distribution of records shifted towards high altitudes within IberBryo. Many Iberian moss survey hotspots are located in classical mountainous survey places, such as the Cantabrian and Sierra Nevada mountain ranges (see Suppl. material 6). Such preference of recorders for mountainous areas and natural reserves has been previously described for other taxa and may be related to the lower human impacts in these areas, their higher diversity due to their typically steeper environmental and habitat gradients or their general attraction for naturalists and the general public (see, for example, Lobo and Martin-Piera 2002, Yang et al. 2014, Meyer et al. 2015, Girardello et al. 2019). In contrast, we found noticeable gaps of information in the south-west of Iberia, the Ebro river basin and the inner plateaus, which should be considered for future moss surveys.

It is remarkable how much the absence of basic information aggravates the general limitations of our database. This evidences the necessity of gathering good quality data, as well as documenting metadata information properly. By an in-depth process of record verification and data-cleaning, we were able to improve the first versions of IberBryo, increasing the amount of data useful for the analysis. Despite these improvements, we found an important problem in the records' metadata. The absence of information on the collecting dates, that affected ca. 42% of the occurrences and prevented us from detecting duplicate records, limited significantly our assessment of inventory completeness (see Hortal et al. 2007). This problem especially affected a particular area of our study, Catalonia and Andorra and, to a much less extent, the northern inner plateau. Thus, we had to exclude one of the most surveyed zones of Iberia from all analyses with the temporal component, preventing any global change analysis that requires information on a key aspect, such as date (see Suppl. material 2). We also found inconsistent dates in some records of the Medina et al. (2015) catalogue during the data curation process. Some years of survey were incorrectly added to the catalogue, based on oral communication with B. Albertos and we needed to search for the original sampling years in the field notes. This implies that a revision of the vouchers and/or field notebooks by the recorders is fundamental to check the actual quality of the available information. These practices could also mobilise a massive amount of data and significantly increase the coverage of high-quality information provided by IberBryo.

Publicly-available Iberian moss records presented other common problems of biodiversity data related to georeferencing (Yesson et al. 2007, Yang et al. 2013, Maldonado et al. 2015, Meyer et al. 2016, Stropp et al. 2016). The absence of geographical coordinates affected ca. 30% of the occurrences in IberBryo v0.1 and the lack or inaccuracy of locality information led to discarding a substantial part. Fortunately, we were able to recover nearly half of these records through geocoding. This process is not often considered in this kind of studies because it is thought to imply an unaffordable effort, but in our case, the improvement obtained was worth the time invested. We also detected taxonomic issues in the GBIF records, although to a lesser extent. These were related to the necessity of taxonomic standardisation of the data and the update of synonymies to currently-accepted names and misidentifications of wrong locations (as, for example, some American species are attributed to our study area). It is also important to mention the absence of substrate and/or habitat type information in many records, which implies the need to acquire it from external references. This prevents the assessment of eventual changes in substrate due to climatic variations, responses to land-use changes or any other ecological effect. The overall knowledge on the ecological responses of moss species would be highly beneficial if this information were added as part of the metadata of their records. The generality of these issues altogether evidences how simple and costless practices of collectors, such as digitising metadata information, could improve the public knowledge of a whole group of organisms, such as bryophytes.

The spatial coverage of Iberian moss surveys through time shows two distinct periods. On the one hand, records follow a patchy distributed pattern until 1970. The surveys showed a remarkable stop in the acquisition of new records between 1935 and 1969 – a setback attributable to the Spanish Civil War and the dictatorships suffered during this period in Spain and Portugal that has been previously described in other groups of organisms (Hortal et al. 2008). However, the overall surveys of the Iberian Peninsula identified many different species – ca. 450 – relatively early (before 1935), which is more than half of the total of species included in the current checklist of IberBryo. The second period shows a clear intensification in moss surveys since 1970, which increased their spatial extent to cover almost the entire Iberian Peninsula. Particularly, after the year 2000, our results show that surveys are concentrated in specific areas where bryophyte research has been more intense (see above), with a limitation in the extent of coverage in several regions that had been moderately well surveyed in the past. This pattern is common in many distributional information, where some well-surveyed areas remain biodiversity hotspots despite lacking recent surveys (see Meyer et al. 2015, Meyer et al. 2016, Stropp et al. 2016) and new surveys come from ecological studies concentrated in particular areas (see below), without following a geographically-stratified sampling design adequate for macroecological studies (Brown 1995, Funk et al. 2005, Hortal and Lobo 2005). However, the quality and usefulness of biodiversity information decays with time due to the unavoidable effects of taxonomic, land use and climatic changes, amongst others (Ladle and Hortal 2013, Tessarolo et al. 2017). This calls for establishing a series of re-surveys of classical localities, which would allow updating the information on these areas, as well as assessing eventual changes in the composition of bryophyte floras.

Interestingly, our findings on spatial coverage at two different cell resolutions allowed us to show that local surveys of mosses are not reflected at regional scale, so well-surveyed areas coincide only partially amongst resolutions (see Suppl. materials 4, 5). Actually, the correlation between survey effort and observed species richness is comparatively lower (0.68) in the 2000-2018 period at finer resolution (Suppl. material 7). Besides, the number of well-surveyed cells does not increase at the coarser spatial resolution, reflecting that heterogeneous and incomplete local inventories could generate reliable regional species inventories under some circumstances. This result is opposite to Lobo et al. (2018) and La Sorte and Somveille (2020), who observed a close similarity of well-sampled areas at different resolutions for other groups. This is likely due to the effect of surveys orientated towards the ecological study of moss communities, where replicates of the same location and/or substrate are desirable (see, for example, Rams 2007, Cezón and Muñoz 2013, Medina et al. 2015, Hespanhol 2017). This is opposite to the typical floristic surveys of former decades, where interest was focused on inventorying as many species and localities as possible. This kind of information on survey trends is lost in higher scales and does not generate well-surveyed areas. In this sense, the assessment of collecting effort at different resolutions can be a good tool to understand the overall quality of the surveys (Oliveira et al. 2016).

Despite all the gaps and biases identified by our study, we find that Iberian climatic gradients — including the rarest climates — are fairly represented by the limited number of well-surveyed 30' cells, which just represent 5% of Iberia. That said, it is clear that it is highly desirable to enlarge the climatic coverage to improve the reliability of any species distribution model or similar approaches that are conducted with these data to assess the effects of climate change, invasions or other aspects of global change (Oliveira et al. 2016). The fact that well-surveyed cells are biased towards anthropised areas would not allow assessing macroecological effects of land-use intensification with fairness. This is despite the opportunity provided by the high density of recent surveys in northern Portugal, where well-surveyed areas are located in an area of intense land-use changes, mainly due to the wood-fires of the last decade. These novel results call for investigating whether these type of biases are general for other regions and biological groups. Additionally, updated information on comparable areas that have not suffered such transformations would be needed to provide a fair evaluation of the effects of this recent land transformation on moss communities, allowing us to assess the impact of global change on this group of organisms.

### Final remarks and future insights

We show that the publicly-available information on Iberian mosses presents significant biases, related to the Wallacean shortfall, but also to basic knowledge on their ecology. This calls for a careful use of this information for biogeographical, ecological modelling and macroecological analysis. It could be argued that the over-representation of certain areas or environments caused by the spatial biases in the data is a relatively minor problem, if overall coverage of climatic and land-use gradients were good. However, opposite to the most intensely-sampled areas, we find noticeable spatial gaps in the information, particularly in the south-west of Iberia and the inner plateaus. The lack of information from these regions compromises any assessment of the processes behind species diversity patterns, as well as the implementation of conservation biogeography approaches (Reddy and Dávalos 2003, Lomolino 2004, Whittaker et al. 2005, Hortal et al. 2007, Hortal et al. 2008). Furthermore, the development of ecological, evolutionary and biogeographical research on Iberian mosses currently requires more surveys with an adequate spatial design and planning (see Hortal and Lobo 2005, Medina et al. 2013). This would maximise their effectiveness, as exemplified by the results of one performed on Iberian epiphytic mosses (Medina et al. 2015). We, therefore, encourage Iberian bryologists to base their future surveys on the information of data gaps provided by the analysis of IberBryo. They could design their surveys using spatially-explicit tools that account for maximising the coverage of the steep environmental and global change that currently characterises the highly dynamic Iberian landscapes. Finally, the limitations associated with incomplete data and metadata could be easily sorted out with improved protocols for data gathering and processing. Further, we are aware that substantial herbarium information may still be waiting for digitalisation and it is not yet accessible through online databases. Beyond reducing the existing biases, enlarging current collections with records from places with poor knowledge outside of the traditionally-surveyed and attractive places will allow us to evaluate the effects of global change on moss communities, leading to both advance knowledge on the ecology and biogeography of Iberian mosses and making informed recommendations for their conservation.

## Supplementary Material

C4029EFC-7EF8-5141-A6F1-7C56F218A51E 10.3897/BDJ.8.e53474.suppl1 Supplementary material 1 IberBryo Database v1.0 Data type Occurrences Brief description IberBryo database (.txt format; UTF-8 encoding)
Also available in: Ronquillo, Cristina; Hortal, Joaquín; 2020; "IberBryo - iberian mosses occurrences dataset"; DIGITAL-CSIC; Version 1.0; http://dx.doi.org/10.20350/digitalCSIC/12494 (This excel version includes fields' descriptions). File: oo_439328.zip https://binary.pensoft.net/file/439328 C. Ronquillo, V. Mazimpaka & J. Hortal

E704DE88-BED9-521F-A53D-2041CA0D8F8F 10.3897/BDJ.8.e53474.suppl2 Supplementary material 2 Distribution Maps of Iberian Moss Occurrences Data type Map Brief description (A) IberBryo v1.1 occurrences (47,730), (B) Preprocessed occurrences without collecting date (34,852) (C) Occurrences from GBIF before data-cleaning and validation process (33,382). File: oo_396081.jpg https://binary.pensoft.net/file/396081 C. Ronquillo

9F1E5F0B-0E04-5DB5-BE45-01642AB66111 10.3897/BDJ.8.e53474.suppl3 Supplementary material 3 Checklist of species included in Iberbryo v1.0 and their frequency in each class of substrate. Data type Table Brief description Frequency of used substrate [1] Rare substrate [2] Occasional substrate [3] Normal substrate. File: oo_401783.xlsx https://binary.pensoft.net/file/401783 C. Ronquillo & N. G. Medina

6694C0BF-192F-5638-A314-64D26F0099D2 10.3897/BDJ.8.e53474.suppl4 Supplementary material 4 Spatial coverage at 5’ resolution. Plates show the number of records in different periods, for the complete time series (IberBryo v1.1) and including records without information on the collecting date (IberBryo v1.0). Data type Map File: oo_396087.jpg https://binary.pensoft.net/file/396087 C. Ronquillo & J. Hortal

1B6B9F37-C04C-5B3D-913E-C49601E73C4E 10.3897/BDJ.8.e53474.suppl5 Supplementary material 5 Spatial coverage of IberBryo v1.1 at 5’ resolution. Plates show the observed richness in different periods, for the complete time series (IberBryo v1.1) and including records without information on the collecting date (IberBryo v1.0). Data type Map File: oo_396088.jpg https://binary.pensoft.net/file/396088 C. Ronquillo & J. Hortal

BFD8ADFF-7673-5D51-824D-F7005586FF9A 10.3897/BDJ.8.e53474.suppl6 Supplementary material 6 Spatial coverage of IberBryo v1.1 at 5’ resolution. Plates show the inventory completeness in different periods, for the complete time series (IberBryo v1.1) and including records without information on the collecting date (IberBryo v1.0). Data type Map File: oo_396089.jpg https://binary.pensoft.net/file/396089 C. Ronquillo & J. Hortal

A01C9FAA-64C3-51B1-8415-960147E0C619 10.3897/BDJ.8.e53474.suppl7 Supplementary material 7 Correlations between records and observed richness per cell. Data type Table File: oo_396090.docx https://binary.pensoft.net/file/396090 C. Ronquillo

CE947B20-7A35-559A-87B6-6C8DD729EB24 10.3897/BDJ.8.e53474.suppl8 Supplementary material 8 Grid cells classified as ‘survey hotspots’ at 30' resolution. Data type Table File: oo_396092.docx https://binary.pensoft.net/file/396092 C. Ronquillo

909D1A9E-8385-5174-8B1A-56559CC7EAFC 10.3897/BDJ.8.e53474.suppl9 Supplementary material 9 Climatic coverage PCA analysis Data type Figure Brief description (A) Distribution of Worldclim 2.0 biovariables at 30’ resolution along the space described by the two climatic axes identified by a PCA. (B) Distribution of Schoener’s D of climatic variability in our study area (grey bars). The dashed red line indicates the Schoener’s D overlap value of well-sampled mosses sites. (C) Geographical distribution of PCA axes scores in the Iberian Peninsula. Colour gradients represent the values of each cell in the corresponding axis, ranging from the most negative (white) to the most positive (green) scores (see the corresponding scale bars). (D) Comparison between the density of PCA scores of the Iberian Peninsula (black line) and the well-surveyed bryophyte cells (red line) for each PCA axis. File: oo_396093.jpg https://binary.pensoft.net/file/396093 C. Ronquillo, F. Alves-Martins & J. Hortal

008E4E21-4382-552B-ACE3-A34CC3751336 10.3897/BDJ.8.e53474.suppl10 Supplementary material 10 Results of the PCA of climatic variables based on WorldClim 2.0 biovariables at 30’ resolution. Data type Table File: oo_396094.docx https://binary.pensoft.net/file/396094 C. Ronquillo, F. Alves-Martins & J. Hortal

D4E5E6AF-3F72-59B5-BEB8-A7FCCEE2E1BF 10.3897/BDJ.8.e53474.suppl11 Supplementary material 11 Reclassifications of land-use categories of CORINE classes used in this work. Data type Table Brief description Reclassification 1 corresponds to aggregated classes of CORINE according to the importance of bryophyte natural history. Reclassification 2 corresponds to whether each type of land-use is (arguably) of artificial or natural origin. File: oo_396095.docx https://binary.pensoft.net/file/396095 C. Ronquillo, F. Alves-Martins & J. Hortal

0A4BFE9E-96D5-5A69-8F13-E07C18958A2A 10.3897/BDJ.8.e53474.suppl12 Supplementary material 12 IberBryo Database Protocol Data type Text Brief description Detailed process of IberBryo creation File: oo_436714.pdf https://binary.pensoft.net/file/436714 C. Ronquillo

05631914-B8FA-5CFE-B4F2-F09C1B669FE5 10.3897/BDJ.8.e53474.suppl13 Supplementary material 13 Coverage analysis R scripts Data type Scripts Brief description The folder contains 3 R scripts used in this work.: 'Climatic coverage analysis' , 'Land use coverage analysis' and 'Temporal and Spatial coverage analysis' File: oo_436716.zip https://binary.pensoft.net/file/436716 C. Ronquillo, F. Alves-Martins, T. Sobral-Souza, B. Vilela-Silva
