# Supplementary figures and images for: Assessing spatial and temporal biases and gaps in the publicly available distributional information of Iberian mosses
Source: Biodivers Data J. 2020 Sep 15;8:e53474. doi: 10.3897/BDJ.8.e53474 (PMC7508938; doi:10.3897/BDJ.8.e53474)

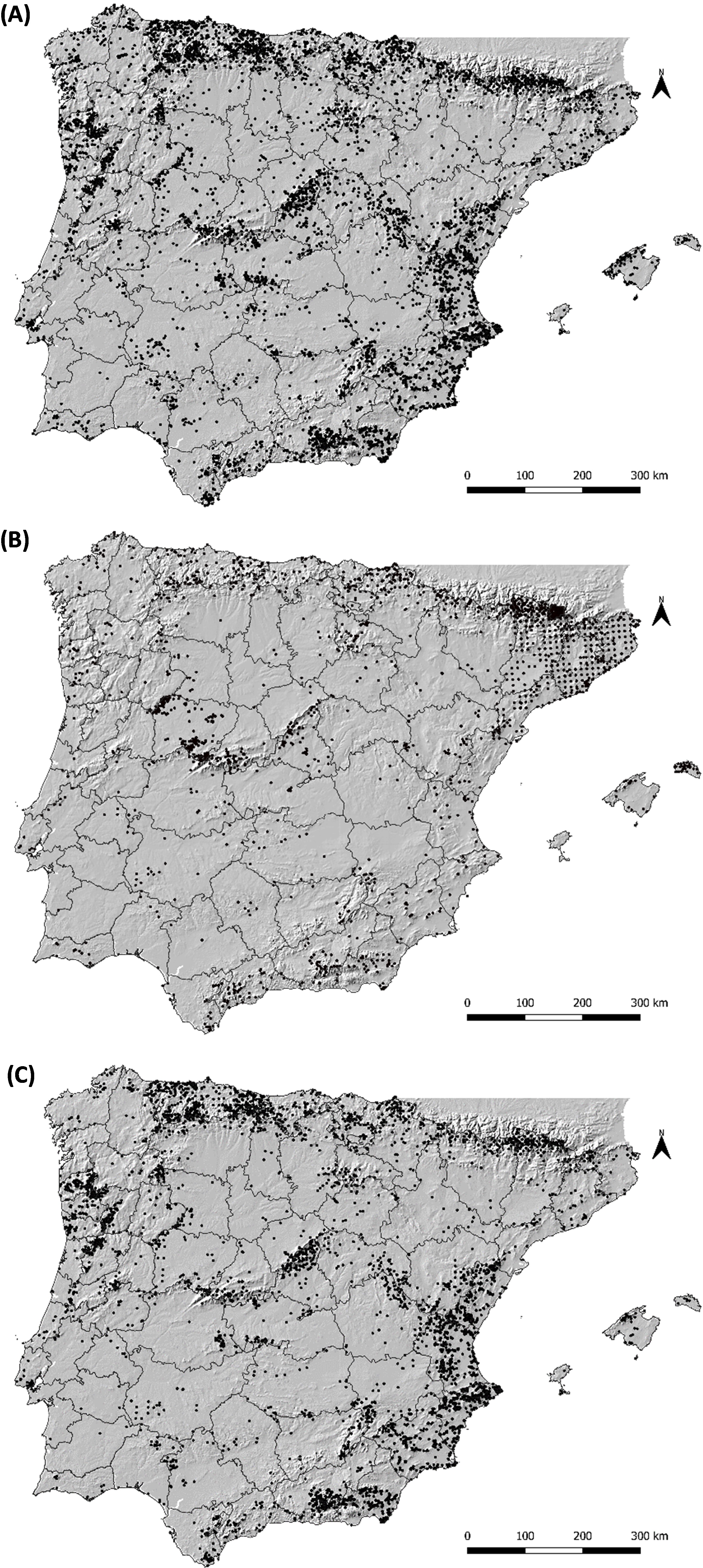

Supplement: Supplementary material 2 — Distribution Maps of Iberian Moss Occurrences [file bdj-08-e53474-s002.jpg]

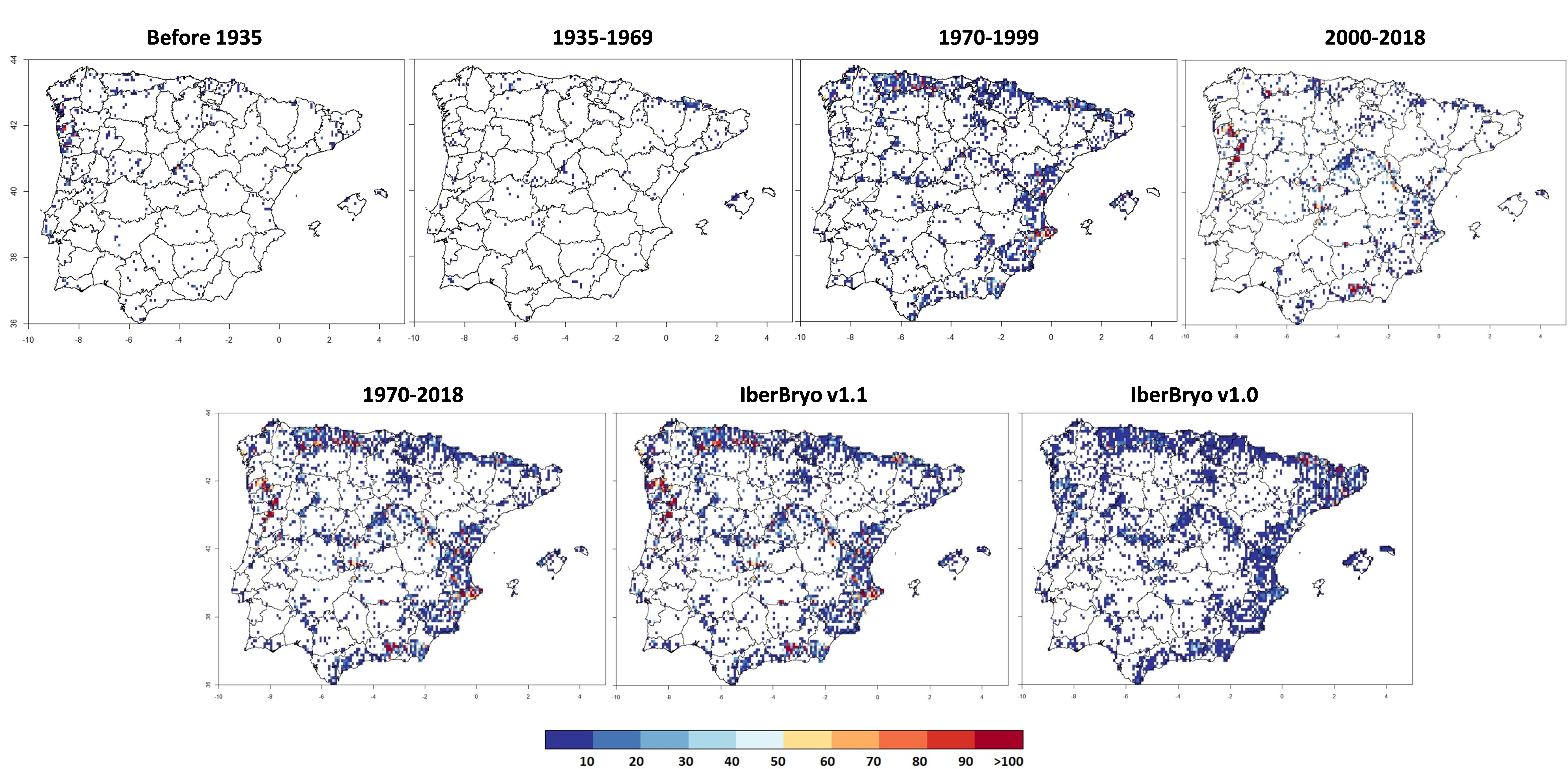

Supplement: Supplementary material 4 — Spatial coverage at 5’ resolution. Plates show the number of records in different periods, for the complete time series (IberBryo v1.1) and including records without information on the collecting date (IberBryo v1.0). [file bdj-08-e53474-s004.jpg]

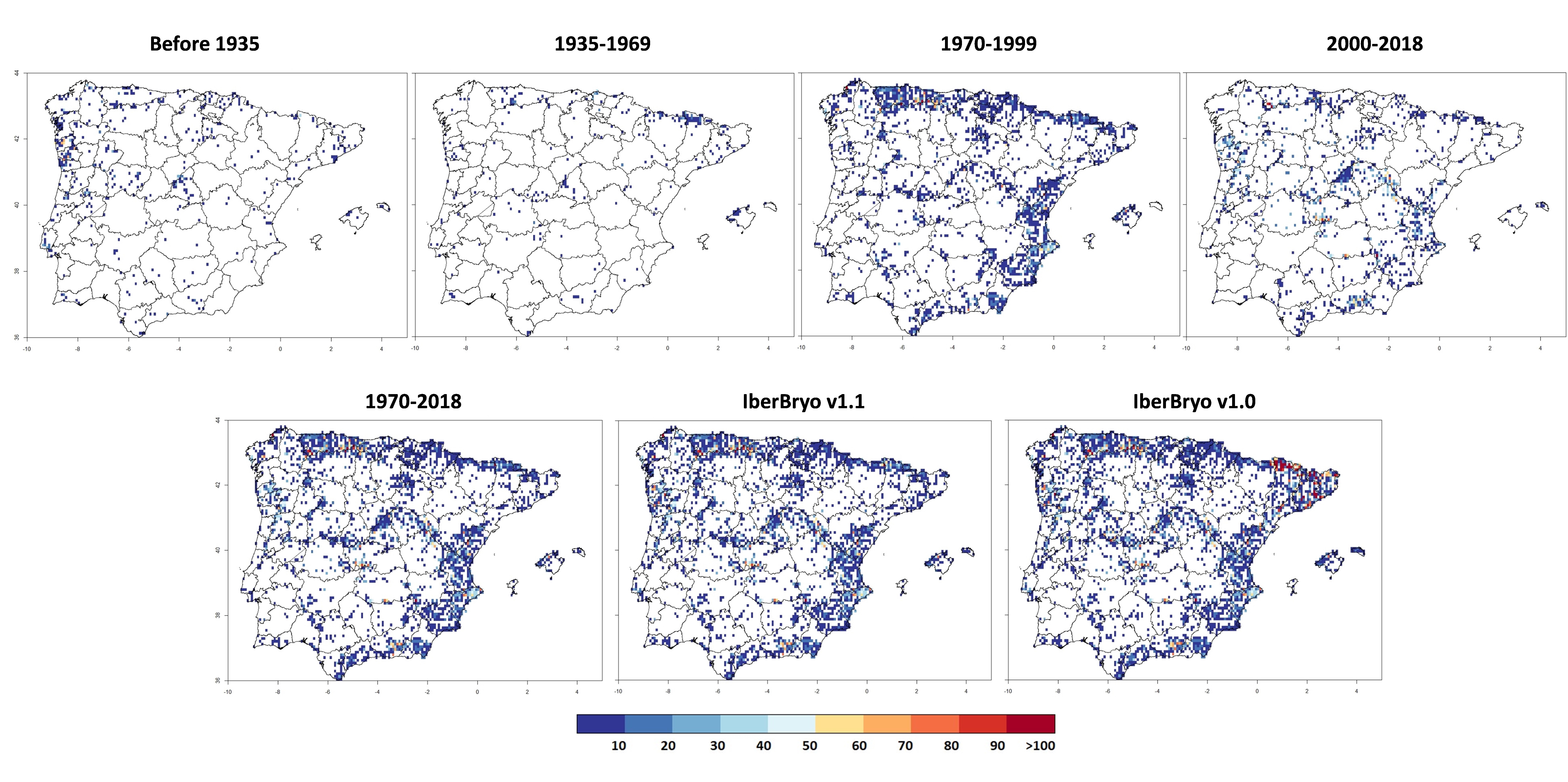

Supplement: Supplementary material 5 — Spatial coverage of IberBryo v1.1 at 5’ resolution. Plates show the observed richness in different periods, for the complete time series (IberBryo v1.1) and including records without information on the collecting date (IberBryo v1.0). [file bdj-08-e53474-s005.jpg]

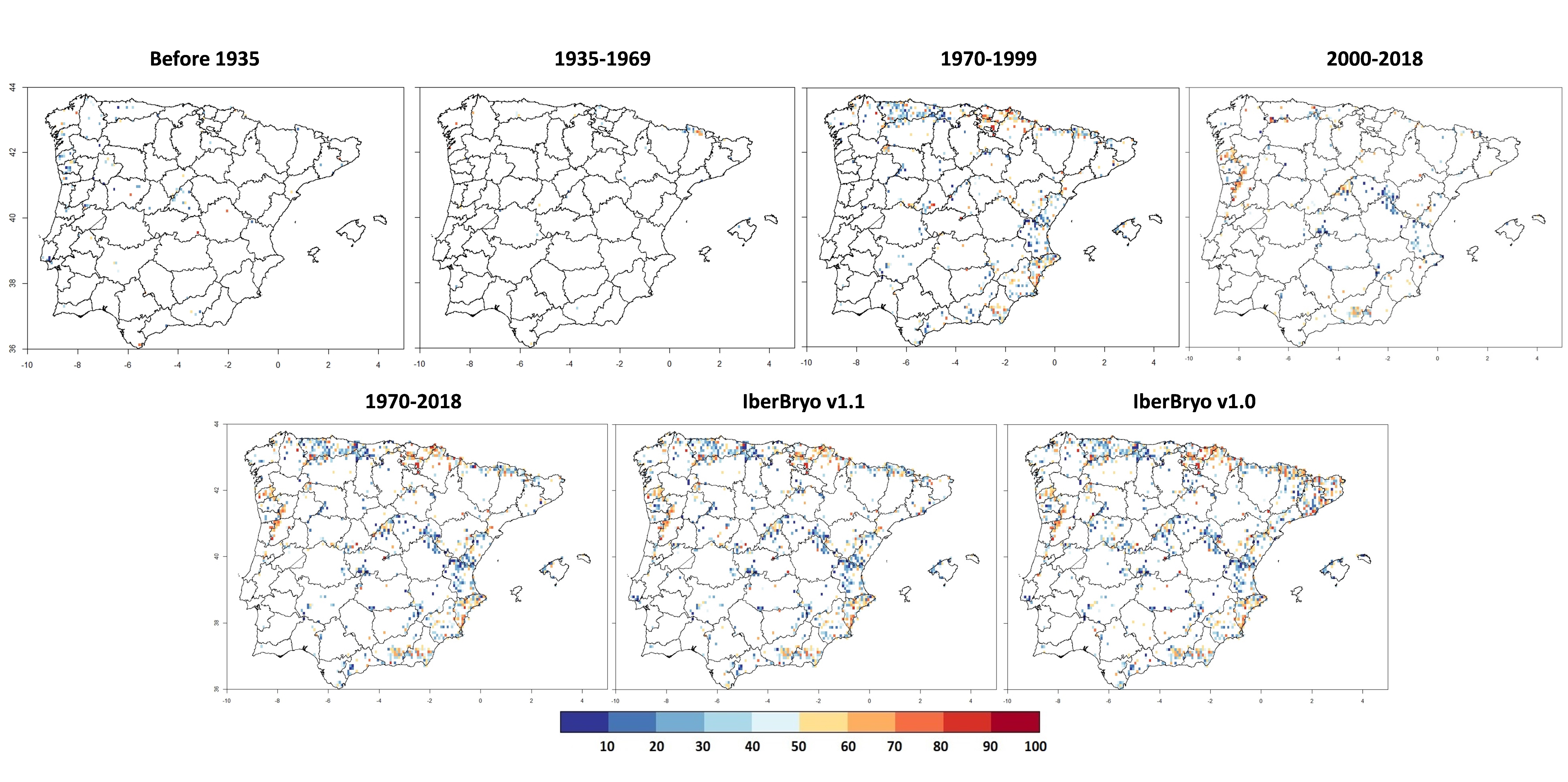

Supplement: Supplementary material 6 — Spatial coverage of IberBryo v1.1 at 5’ resolution. Plates show the inventory completeness in different periods, for the complete time series (IberBryo v1.1) and including records without information on the collecting date (IberBryo v1.0). [file bdj-08-e53474-s006.jpg]

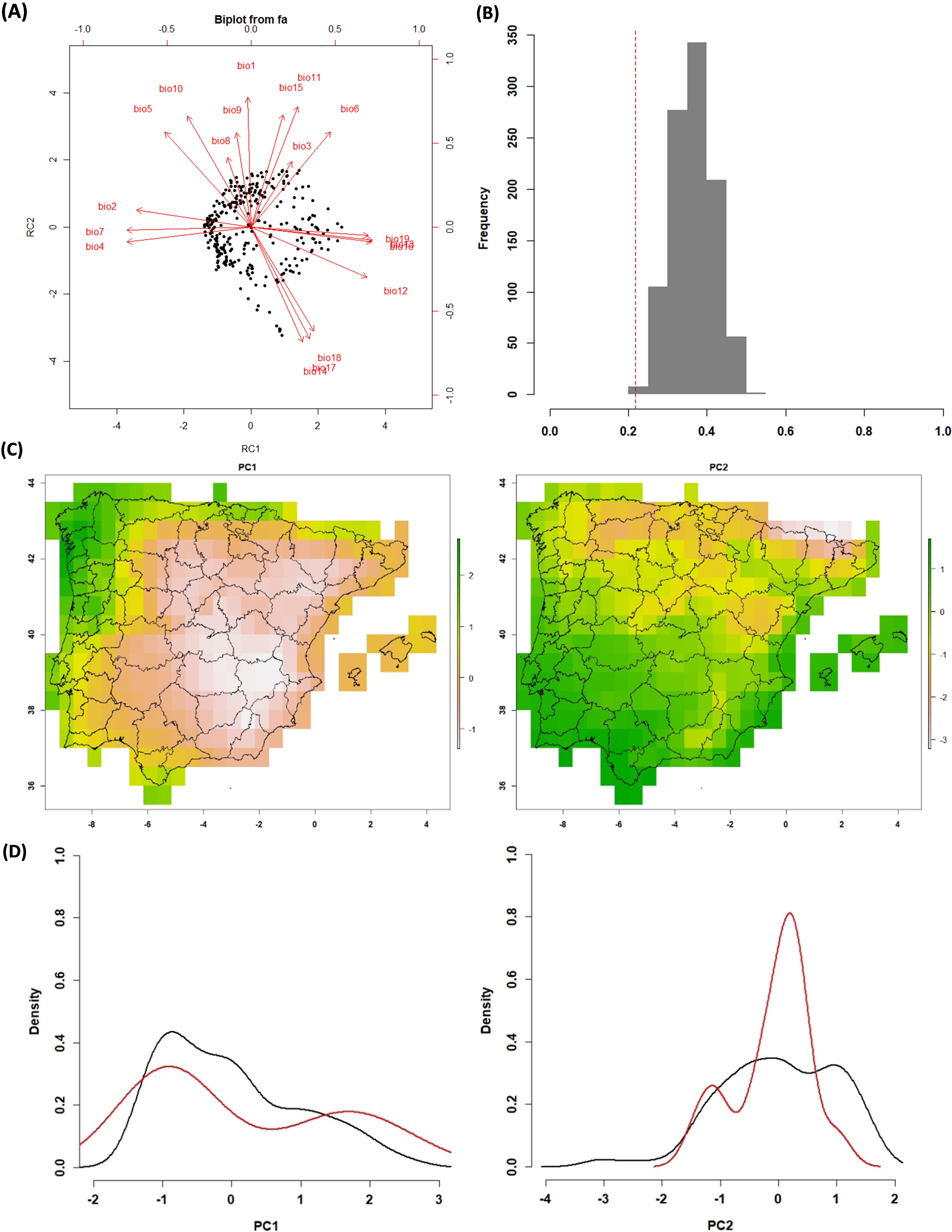

Supplement: Supplementary material 9 — Climatic coverage PCA analysis [file bdj-08-e53474-s009.jpg]
