# Supplementary material for: Assessing spatial and temporal biases and gaps in the publicly available distributional information of Iberian mosses
Source: Biodivers Data J. 2020 Sep 15;8:e53474. doi: 10.3897/BDJ.8.e53474 (PMC7508938; doi:10.3897/BDJ.8.e53474)
Supplement: Supplementary material 7 — Correlations between records and observed richness per cell. [file bdj-08-e53474-s007.docx]

**Table S7.** Correlations between records and observed richness per cell.

| **Resolution cell** | **Before 1935** | **1935-1969** | **1970-1999** | **2000-2018** | **1970-2018** | **IberBryo v1.1** | **IberBryo v1.0** |
| --- | --- | --- | --- | --- | --- | --- | --- |
| **5’** | 0.98 | 0.97 | 0.94 | 0.68 | 0.74 | 0.74 | 0.84 |
| **30’** | 0.97 | 0.98 | 0.86 | 0.83 | 0.83 | 0.85 | 0.83 |
