# Supplementary material for: Assessing spatial and temporal biases and gaps in the publicly available distributional information of Iberian mosses
Source: Biodivers Data J. 2020 Sep 15;8:e53474. doi: 10.3897/BDJ.8.e53474 (PMC7508938; doi:10.3897/BDJ.8.e53474)
Supplement: Supplementary material 8 — Grid cells classified as ‘survey hotspots’ at 30' resolution. [file bdj-08-e53474-s008.docx]

**Table S8.** 30' grid cells classified as ‘survey hotspots’.

| **Time period** | **Place** | **Number of records** | **Observed richness** |
| --- | --- | --- | --- |
| 1970-1999 | Cantabrian Mountains | 1483 | 275 |
| 1970-1999 | Cantabrian Mountains | 950 | 245 |
| 1970-1999 | Cantabrian Mountains | 882 | 280 |
| 1970-1999 | Valencia | 1344 | 140 |
| 2000-2018 | Sierra Nevada | 1517 | 182 |
| 2000-2018 | Cantabrian Mountains | 1995 | 188 |
| 2000-2018 | Northern Portugal | 1125 | 94 |
| 2000-2018 | Northern Portugal | 1223 | 116 |
| No info on date | Northern Plateau | 745 | 132 |
| No info on date | Northern Plateau | 746 | 150 |
| No info on date | Northern Plateau | 878 | 169 |
| No info on date | Catalonia | 6282 | 430 |
| No info on date | Catalonia | 6186 | 373 |
| No info on date | Catalonia | 2977 | 297 |
| No info on date | Catalonia | 2639 | 335 |
| No info on date | Catalonia | 2436 | 297 |
