# Supplementary material for: Assessing spatial and temporal biases and gaps in the publicly available distributional information of Iberian mosses
Source: Biodivers Data J. 2020 Sep 15;8:e53474. doi: 10.3897/BDJ.8.e53474 (PMC7508938; doi:10.3897/BDJ.8.e53474)
Supplement: Supplementary material 10 — Results of the PCA of climatic variables based on WorldClim 2.0 biovariables at 30’ resolution. [file bdj-08-e53474-s010.docx]

**Table S10.** Results of the PCA of climatic variables based on WorldClim 2.0 biovariables at 30’ resolution.

|  | RC1 | RC2 | h2 | u2 | com |
| --- | --- | --- | --- | --- | --- |
| wc2.0_10m_1 | -0.02 | 0.97 | 0.93 | 0.065 | 1.0 |
| wc2.0_10m_10 | -0.47 | 0.83 | 0.91 | 0.090 | 1.6 |
| wc2.0_10m_11 | 0.35 | 0.90 | 0.93 | 0.074 | 1.3 |
| wc2.0_10m_12 | 0.86 | -0.38 | 0.88 | 0.116 | 1.4 |
| wc2.0_10m_13 | 0.90 | -0.10 | 0.82 | 0.176 | 1.0 |
| wc2.0_10m_14 | 0.38 | -0.86 | 0.88 | 0.122 | 1.4 |
| wc2.0_10m_15 | 0.24 | 0.84 | 0.76 | 0.241 | 1.2 |
| wc2.0_10m_16 | 0.90 | -0.11 | 0.82 | 0.178 | 1.0 |
| wc2.0_10m_17 | 0.44 | -0.83 | 0.88 | 0.117 | 1.5 |
| wc2.0_10m_18 | 0.47 | -0.78 | 0.82 | 0.179 | 1.6 |
| wc2.0_10m_19 | 0.87 | -0.06 | 0.77 | 0.234 | 1.0 |
| wc2.0_10m_2 | -0.85 | 0.13 | 0.74 | 0.260 | 1.0 |
| wc2.0_10m_3 | 0.31 | 0.49 | 0.33 | 0.668 | 1.7 |
| wc2.0_10m_4 | -0.92 | -0.11 | 0.87 | 0.134 | 1.0 |
| wc2.0_10m_5 | -0.64 | 0.71 | 0.91 | 0.091 | 2.0 |
| wc2.0_10m_6 | 0.59 | 0.71 | 0.86 | 0.144 | 1.9 |
| wc2.0_10m_7 | -0.92 | -0.02 | 0.85 | 0.147 | 1.0 |
| wc2.0_10m_8 | -0.18 | 0.52 | 0.30 | 0.699 | 1.2 |
| wc2.0_10m_9 | -0.11 | 0.70 | 0.51 | 0.493 | 1.0 |

|  | RC1 | RC2 |
| --- | --- | --- |
| SS loadings | 7.42 | 7.35 |
| Proportion Var | 0.39 | 0.39 |
| Cumulative Var | 0.39 | 0.78 |
| Proportion Explained | 0.50 | 0.50 |
| Cumulative Proportion | 0.50 | 1.00 |

Mean item complexity = 1.3

Test of the hypothesis that 2 components are sufficient.

The root mean square of the residuals (RMSR) is 0.1

with the empirical chi square 1141.74 with prob < 2.1e-159

Fit based upon off diagonal values = 0.96
