# Supplementary material for: Assessing spatial and temporal biases and gaps in the publicly available distributional information of Iberian mosses
Source: Biodivers Data J. 2020 Sep 15;8:e53474. doi: 10.3897/BDJ.8.e53474 (PMC7508938; doi:10.3897/BDJ.8.e53474)
Supplement: Supplementary material 11 — Reclassifications of land-use categories of CORINE classes used in this work. [file bdj-08-e53474-s011.docx]

**Table S11.** Reclassifications of land-use categories of CORINE classes used in this work. Reclassification 1 corresponds to aggregated classes of CORINE according to the importance of bryophyte natural history. Reclassification 2 corresponds to whether each type of land-use is (arguably) of artificial or natural origin.

| **CORINE Class** | **Description** | **Reclassification 1** | **Reclassification 2** |
| --- | --- | --- | --- |
| **1** | **Artificial Surfaces** |  |  |
| 111 | Continuous urban fabric | 1 | Artificial |
| 112 | Discontinuous urban fabric | 1 | Artificial |
| 121 | Industrial or commercial units | 1 | Artificial |
| 122 | Road and rail networks and associated land | 1 | Artificial |
| 123 | Port areas | 1 | Artificial |
| 124 | Airports | 1 | Artificial |
| 131 | Mineral extraction sites | 1 | Artificial |
| 132 | Dump sites | 1 | Artificial |
| 133 | Construction sites | 1 | Artificial |
| 141 | Green urban areas | 1 | Artificial |
| 142 | Sport and leisure facilities | 1 | Artificial |
| **2** | **Agricultural Areas** |  |  |
| 211 | Non-irrigated arable land | 2 | Artificial |
| 212 | Permanently irrigated land | 2 | Artificial |
| 213 | Rice fields | 2 | Artificial |
| 221 | Vineyards | 2 | Artificial |
| 222 | Fruit trees and berry plantations | 2 | Artificial |
| 223 | Olive groves | 2 | Artificial |
| 231 | Pastures | 4 | Natural |
| 241 | Annual crops associated with permanent crops | 2 | Artificial |
| 242 | Complex cultivation patterns | 2 | Artificial |
| 243 | Land principally occupied by agriculture | 2 | Artificial |
| 244 | Agro-forestry areas | 5 | Natural |
| **3** | **Forest and semi-natural areas** |  |  |
| 311 | Broad-leaved forest | 3 | Natural |
| 312 | Coniferous forest | 3 | Natural |
| 313 | Mixed forest | 3 | Natural |
| 321 | Natural grasslands | 4 | Natural |
| 322 | Moors and heathland | 6 | Natural |
| 323 | Sclerophyllous vegetation | 7 | Natural |
| 324 | Transitional woodland-shrub | 7 | Natural |
| 331 | Beaches, dunes, sands | 8 | Natural |
| 332 | Bare rocks | 9 | Natural |
| 333 | Sparsely vegetated areas | 7 | Natural |
| 334 | Burnt areas | 10 | Artificial |
| 335 | Glaciers and perpetual snow | 3 | Natural |
| **4** | **Wetlands** |  |  |
| 411 | Inland marshes | 11 | Natural |
| 412 | Peat bogs | 11 | Natural |
| 421 | Salt marshes | 12 | Natural |
| 422 | Salines | 12 | Natural |
| 423 | Intertidal flats | 12 | Natural |
| **5** | **Water bodies** |  |  |
| 511 | Water courses | 11 | Natural |
| 512 | Lakes | 11 | Natural |
| 521 | Coastal lagoons | 12 | Natural |
| 522 | Estuaries | 12 | Natural |
| 523 | Sea and ocean | 12 | Natural |
