# Supplementary material for: Assessing spatial and temporal biases and gaps in the publicly available distributional information of Iberian mosses
Source: Biodivers Data J. 2020 Sep 15;8:e53474. doi: 10.3897/BDJ.8.e53474 (PMC7508938; doi:10.3897/BDJ.8.e53474)
Supplement: Supplementary material 12 — IberBryo Database Protocol [file bdj-08-e53474-s012.pdf]

IberBryo database protocol

1) **Download** data from the Official website of GBIF(https://www.gbif.org/occurrence/search):

- Log in
- Add filters:
  - Phylum = ‘Bryophyta’
  - Country = ‘Spain’, ‘Portugal’, ‘Andorra’, ‘Gibraltar’
- Download *Simple* csv format.

2) **Geospatial validation**

a. Data preparation (Software – Microsoft Excel):

i. Add manually not duplicated records provided in Cezón & Muñoz (2013) and Medina et al. (2015) dissertations.

Medina et al. (2015) is now available in GBIF so it is not needed to add their records to GBIF downloads anymore:

Medina N, Ronquillo Ferrero C (2020). Epiphytic mosses from the northwest Iberian quadrant (Spain). Spanish National Museum of Natural Sciences (CSIC). Occurrence dataset https://doi.org/10.15470/rqv6jb accessed via GBIF.org

ii. Split the dataset into 2 subsets: ‘not available coordinates’ (‘decimalLongitude’ & ‘decimalLatitude’ fields filtered = ‘0’ or ‘empty’) and ‘available coordinates’.

b. ‘Available coordinates’ subset processing:

i. ‘*Point in polygon test*’: Check whether available coordinates are located within the administrative boundaries at county level based on the information provided in ‘county’ field (Software – QGIS 3.4 and Microsoft Excel)

1. Add the records to QGIS software as *Delimited Text*.
2. Add the administrative map of the study area – defined as mainland Portugal and Spain, plus the Balearic Islands, Andorra and Gibraltar – from *GADM maps and data* (https://gadm.org/) at level 0 for country validation and level 2 for province validation.
3. In QGIS: Process → ToolBox → Search and click: ‘Add polygon attributes to point’ from SAGA.
   - Points = Records shapefile

- Polygons = GADM administrative boundaries
- Attribute = ‘NAME_0’ (Name of the country)

  This process adds a new column in the Attribute Table with the name of the administrative unit where coordinates are located.

4. Repeat the process with the result shapefile as Points and change the attribute argument to ‘Name_2’ (Name of the county) and for each one of the countries of the study area.
5. Export the final ‘Result’ shapefile as CSV.
6. Open the file (Excel) and manually validate through filters whether the records are:

   a. In the same country and county/province as ‘country_Code’, ‘county’, and ‘locality’ fields information. *Validated records*

   b. Outside the country or county/province.

   1) Check manually *typos* in provided coordinates:
      a. Swap longitude/latitude.
      b. Numerical sign errors near Prime Meridian.
   2) Delete records if no correction is possible.

   c. If ‘Name_0’ field is empty the record is classified as ‘Sea coordinates’.

ii. ‘Sea coordinates’ subset validation:

1. (Software – R /RStudio) Assign new coordinates to the nearest coastal place in a range of 10 km (based on the size of the grid cells analysis). Delete records that do not meet the criteria.
   #Package ‘seegSDM’ function NearestLand (Based on WorldClim 5 min mask)

   Nick Golding & Freya Shearer (2019). seegSDM: Streamlined Functions for Species Distribution Modelling in the SEEG Research Group. R package

   version 0.1-9.

2. (Software – QGIS 3.4 and Microsoft Excel) Validate the new assigned coordinates through *“point in polygon”* test previously described and the ‘Name placed’ information provided in ‘locality’ field.

c. **'Not available coordinates' subset processing:**

i. Missing 'locality' information – *Delete records*

ii. Geocoding through 'Name placed' information in 'locality' field:

1. Assign coordinates with official gazetteers.

   Required official gazetteers:

   - "Nomenclátor de Municipios y Entidades de población" and "Nomenclátor Básico" of Instituto Geográfico Nacional (IGN) for Spanish records. Available in http://centrodedescargas.cnig.es/CentroDescargas/catalogo.do?Serie=NGBES
   - "Servicio de Localizaçao Toponímica - Grupo Crise Rede de Informaçao de Situações de Emergencia" for Portuguese records. Available in http://scrif.igeo.pt/asp/toponim.asp

   They provide Gaus-Militar XY coordinates that need a one by one transformation through their own website available in http://scrif.igeo.pt/asp/coordenadas/main.asp

   Check option: *Tipo de transformaçao 'XY para WGS84'*

   Degrees (°), minutes ('), seconds ('') WGS84 output is then transformed into decimal coordinates format in https://epsg.io/

   - "Nomenclàtor Oficial del Govern d'Andorra" for Andorran records. Available in https://www.cartografia.ad/nomenclator

2. Delete records with inaccurate locality information: 'Name placed' information corresponding to equal or higher level than county name.

3) Taxonomic validation (Software – Microsoft Excel)

a. Check whether exists enough taxon rank identification: delete records with empty GBIF 'specificEpithet' field.
b. Check fossil species (GBIF 'basisOfRecord' field = FossilSpecimen) and delete them
c. Select all the species name of the dataset included in 'scientificName', 'genus' and 'specificEpithet' fields to create a list of names to check (Delete duplicates).
d. Unification of the species names:

   i. Check *typos*, misspells and scientific name authority variations.

ii. Check synonyms and update scientific names.

Records were reviewed following the checklists in Casas et al. (2006), Hill et al. (2006), Ros et al. (2013), Hodgets (2015), Sotiaux & Vanderporten (2017) and Flora Briofítica Ibérica (Guerra et al., 2006, 2010, 2014, 2018; Brugués et al., 2007; Brugués & Guerra 2015). For the assignation of the species name, we gave priority to the most recent checklist, except for taxa that have furtherly experienced taxonomic or nomenclatural changes: e.g., Bartramia stricta (Müller 2014), Orthotrichum (Plásek et al., 2015; Lara et al., 2016), Codonoblepharon forsteri (Goffinet et al. 2004, Mazimpaka & Lara, 2014), and Oxystegus tenuirostris (Alonso et al. 2016, 2018).

e. Check whether the species are formally cited in the area (at country level). This information is provided in Ros et al. (2013). Delete records that are misplaced.

4) Date validation

Check if it exists information of collecting date filtering records with information in 'year' field (Software – Microsoft Excel).
